# Adipokines Mediate Inflammation and Insulin Resistance

**DOI:** 10.3389/fendo.2013.00071

**Published:** 2013-06-12

**Authors:** Hyokjoon Kwon, Jeffrey E. Pessin

**Affiliations:** ^1^Department of Medicine and Molecular Pharmacology, Albert Einstein College of Medicine, Bronx, NY, USA

**Keywords:** adipokine, adipocyte, inflammation, insulin, macrophages and metabolism

## Abstract

For many years, adipose tissue was considered as an inert energy storage organ that accumulates and stores triacylglycerols during energy excess and releases fatty acids in times of systemic energy need. However, over the last two decades adipose tissue depots have been established as highly active endocrine and metabolically important organs that modulate energy expenditure and glucose homeostasis. In rodents, brown adipose tissue plays an essential role in non-shivering thermogenesis and in energy dissipation that can serve to protect against diet-induced obesity. White adipose tissue collectively referred too as either subcutaneous or visceral adipose tissue is responsible for the secretion of an array of signaling molecules, termed adipokines. These adipokines function as classic circulating hormones to communicate with other organs including brain, liver, muscle, the immune system, and adipose tissue itself. The dysregulation of adipokines has been implicated in obesity, type 2 diabetes, and cardiovascular disease. Recently, inflammatory responses in adipose tissue have been shown as a major mechanism to induce peripheral tissue insulin resistance. Although leptin and adiponectin regulate feeding behavior and energy expenditure, these adipokines are also involved in the regulation of inflammatory responses. Adipose tissue secretes various pro- and anti-inflammatory adipokines to modulate inflammation and insulin resistance. In obese humans and rodent models, the expression of pro-inflammatory adipokines is enhanced to induce insulin resistance. Collectively, these findings have suggested that obesity-induced insulin resistance may result, at least in part, from an imbalance in the expression of pro- and anti-inflammatory adipokines. Thus we will review the recent progress regarding the physiological and molecular functions of adipokines in the obesity-induced inflammation and insulin resistance with perspectives on future directions.

## Introduction

Excess nutrition and sedentary lifestyle induce excessive lipid accumulation in adipose and peripheral tissues resulting in obesity. Obesity has become a pandemic health problem in which more than 60% of American adults are overweight or obese and is closely associated with metabolic diseases such as insulin resistance, type 2 diabetes (T2D), hypertension, non-alcoholic fatty liver disease, and polycystic ovarian diseases (Finkelstein et al., 2012). Thus the financial cost to manage obesity and related diseases is a burden on public healthcare system in modern society. T2D is a quickly growing global metabolic disease characterized by impaired insulin secretion from pancreatic β cells and insulin resistance in liver, muscle, and adipose tissue. In T2D, pancreatic β cells are continuously activated to synthesize and secret insulin due to unresolved hyperglycemia, and this cellular stress gradually induces deterioration and apoptosis of pancreatic β cells (Butler et al., 2003; Ashcroft and Rorsman, 2012). Thus both impaired pancreatic β cell function and insulin resistance further deteriorate physiological consequences of T2D. Muscle and adipocytes show impaired insulin-stimulated glucose uptake with reduced inhibition of liver glucose production. This constellation of tissue specific pathophysiology results in increased fasting glucose levels and the inability to adequately clear glucose from the circulation in the post-prandial state.

The molecular mechanisms of obesity-associated T2D are still unclear, however recent studies have shown that low-grade chronic inflammation is an important factor in the pathogenesis of T2D in humans and rodent animal models (Hotamisligil, 2006; Shoelson et al., 2006; Schenk et al., 2008; Ouchi et al., 2011). Although liver and muscle show obesity-induced mild inflammatory responses without significant numeric changes of immune cells, adipose tissue depots are the most vulnerable target to mediate significant immune cells infiltration and inflammation contributing to systemic inflammation and insulin resistance in obese rodents and humans (Odegaard and Chawla, 2013). Adipose tissue is a major tissue to provide excess nutrient storage for triacylglycerols and also produces various secreted proteins called adipokines as any other bonafide endocrine organ (Waki and Tontonoz, 2007). Adipose tissues produce leptin and adiponectin to regulate feeding behavior and also generate pro- and anti-inflammatory adipokines to modulate inflammatory responses. Adipocytes, the most abundant cell population of adipose tissue, provides reversible excess energy storage depot in adipose tissue. Thus excess nutrition overload initiates adipocytes hypertrophy and hyperplasia resulting in cellular stress that in turn initiates oxidative stress and inflammatory responses in adipose tissue. Inflammatory responses in adipose tissues become self-generating that eventually leads to increased local and systemic levels of various pro-inflammatory cytokines including tumor necrosis factor-α (TNF-α), interleukin-6 (IL-6), IL-1β, and CC-chemokine ligand 2 (CCL2) that are causative for insulin resistance. Along with inflammatory adipokine production in adipose tissues, obesity-related hyperlipidemia, hyperglycemia, hypoxia, oxidative stress, and endoplasmic reticulum (ER) stress can also induce insulin resistance in peripheral tissues and can induce activation of inflammatory signaling cascades in adipose tissues.

Currently a major objective in field is to understand the initiating factors responsible for induction of adipose tissue inflammation and the complex cascade of feed-forward and feed-back mechanism that continue to amplify and maintain the pro-inflammatory state. Only with an understanding of the cellular and molecular crosstalk between adipocytes and the immune system will be able to develop specific therapies to prevent inflammation and restore insulin sensitivity in an effective manner without inducing secondary complications such as ectopic lipid accumulation or further exacerbating obesity. In this review we will focus on the recent progress regarding the physiological and molecular functions of adipokines in the obesity-induced inflammation and insulin resistance.

## Insulin Resistance

The pancreas is primarily a dual function organ composed of exocrine cells that secrete digestive enzymes into the gastrointestinal lumen and endocrine cells localized to the Islets of Langerhans that secrete hormones into the circulation to regulate metabolic processes. Pancreatic islets produce several key endocrine hormones such as insulin, glucagon, and somatostatin necessary for the maintenance of normoglycemia. In particular, insulin secretion is enhanced in response to increased circulating glucose and amino acids. In peripheral tissues, insulin stimulates glucose uptake (skeletal muscle and adipose tissue), glycogen storage (skeletal muscle, liver), and inhibits gluconeogenesis and glycogenolysis (liver). Insulin also increases lipogenesis in hepatocytes and adipocytes and diminishes adipocyte free fatty acid generation from triacylglycerols (lipolysis) (Pessin and Saltiel, 2000). Thus the definition of insulin resistance is the perturbation of insulin-mediated signaling pathway resulting in systemic hyperglycemia. As insulin has pleiotropic functions, insulin resistance is closely linked with other metabolic symptoms such as hypertension and hyperlipidemia (Cornier et al., 2008).

**Figure 1:**
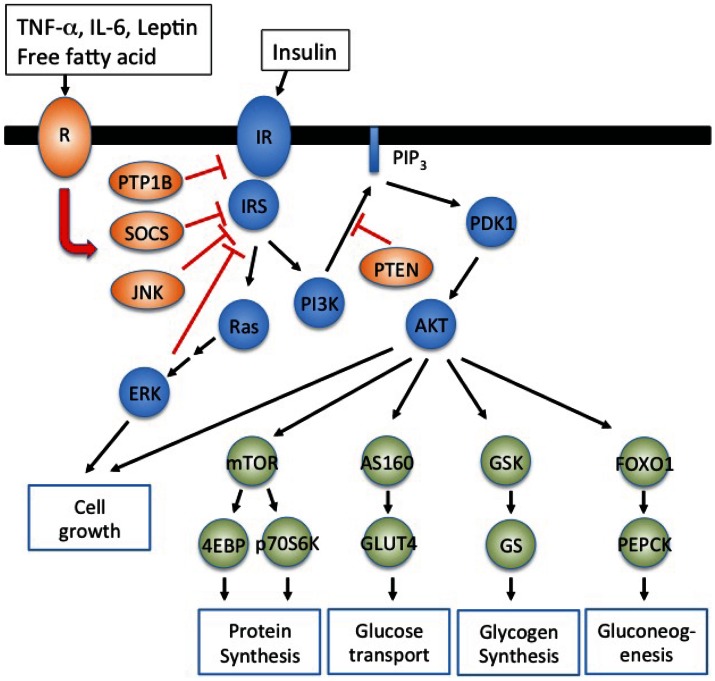
**Inflammatory adipokines suppress insulin signaling resulting in insulin resistance**. IRS1/2 phosphorylated on specific tyrosine residues activates the phosphatidylinositol 3-kinase (PI3K)-AKT/protein kinase B (PKB) pathway and Ras-mitogen-activated protein kinase (MAPK) pathway. PI3K-AKT signaling pathway regulates metabolic processes such as glucose uptake (muscle and adipocytes), glycogen synthesis (muscle and liver), protein synthesis (muscle and liver), and gluconeogenesis (liver). Inflammatory signals, TNF-α, IL-6, LPS, and saturated free fatty acid, activate inhibitory molecules such as SOCS and JNK to suppress insulin signaling resulting in insulin resistance. PI3K dependent PDK1 activation is negatively regulated by phospholipid phosphatases such as phosphatase and tensin homolog (PTEN) that degrade PIP_3_.

To understand insulin resistance, we need to clarify molecular mechanisms of insulin signaling. At the molecular level, insulin binds to the cell surface insulin receptor that exists as an α_2_β_2_ heterodimer (Taniguchi et al., 2006). Following insulin binding the tyrosine kinase domain of β subunits autophosphorylates themselves in a trans-phosphorylation reaction that activates its intrinsic kinase activity to proximal substrates such as insulin receptor substrate (IRS) family (IRS1-IRS4), Src-homology-2-containing (Shc) adaptor proteins, signal-regulatory protein (SIRP) family, and Grb2-associated binder-1 (Gab1). IRS1/2 phosphorylated on specific tyrosine residues activates two major signaling pathways; (i) the phosphatidylinositol 3-kinase (PI3K)-AKT/protein kinase B (PKB) pathway to modulate most metabolic functions of insulin such as glucose transport, glycogen synthesis, gluconeogenesis, protein synthesis, and cell growth and (ii) Ras-mitogen-activated protein kinase (MAPK) pathway (Figure 1). In addition, there are inhibitory molecules for insulin signaling such as the protein tyrosine phosphatase 1B (PTP1B), the suppressor of cytokine signaling (SOCS) and the growth factor receptor bound protein 10 (Grb10) that suppress insulin signaling by inducing insulin receptor dephosphorylation, physical blocking of substrate phosphorylation, and degradation of the insulin receptor and/or IRS. AKT phosphorylates the AKT substrate of 160 kDa (AS160) to activate Rab small GTPase that initiates the translocation of the glucose transporter 4 (GLUT4) resulting in the glucose uptake in muscle and adipocytes. AKT also suppresses glycogen synthase kinase-3 (GSK3) to activate glycogen synthase resulting in the glycogen synthesis in muscle and liver (Cross et al., 1995). The AKT phosphorylation of forkhead box O1 (FOXO1) induces FOXO1 association with 14-3-3 protein, that in turn excludes FOXO1 from the nucleus. In the liver, this suppresses gluconeogenic gene expression and thereby inhibits hepatic glucose output. AKT phosphorylates tuberous sclerosis complex 1 and 2 (TSC1/2), which release the inhibition of Ras homolog enriched in brain (Rheb) for the activation of mTORC1 complex, that in turn enhances protein synthesis through the activation of eukaryotic translation initiation factor 4E binding protein-1 (4E-BP) and p70 ribosomal protein S6 kinase 1 (p70S6K1).

Although insulin signaling is well studied, the molecular mechanisms how insulin resistance develops are still unclear. Alterations in insulin receptor expression, ligand binding, phosphorylation, and kinase activity affect the downstream of insulin signaling resulting in diverse clinical syndromes such as the type A syndrome, leprechaunism, and Rabson–Mendenhall syndrome. Insulin receptor gene (*INSR*) mutations are very rare but at least more than 30 *INSR* mutations have been shown to mediate insulin receptor dysfunction, and these mutations may induce insulin resistance with polygenic defects in its downstream signaling (Hegele, 2003). In addition, mutations of DM1 kinase gene causes defective alternative splicing of *INSR* (Savkur et al., 2001), and mutations of high-mobility group A1 (HMGA1) gene suppress the expression of *INSR* resulting in insulin resistance (Chiefari et al., 2011).

Impaired proximal signaling of insulin receptor also mediates insulin resistance. Decreased IRS protein levels contribute insulin resistance in rodents and humans (Shimomura et al., 2000). A complete molecular understand and mechanisms of reduced IRS levels are still under investigation. However, excess insulin suppresses the expression of IRS2, and SOCS1/3 induced by inflammatory adipokines such as TNF-α, IL-6, and IL-1β enhance the degradation of IRS1/2 through E3 ubiquitin ligase activation (Rui et al., 2002) (Figure 1). IRS phosphorylation on serine residues is another mechanism to induce insulin resistance. IRS contains several serine residues that are phosphorylated by kinases such as extracellular signal regulated kinase (ERK), cJun N-terminal kinase (JNK), protein kinase Cζ (PKCζ), and p70S6K (Boura-Halfon and Zick, 2009). The phosphorylation of IRS on Ser-307 is a typical inhibitory signal to suppress insulin signaling as Ser-307 locates in PTB domain of IRS (Hirosumi et al., 2002). Thus increased TNF-α and saturated free fatty acids in obese individuals activate JNK and inhibitor of nuclear factor κB kinase β (IKKβ) to phosphorylate Ser-307 of IRS. In addition ERK activated by insulin also phosphorylates IRS1 on Ser-612 to attenuate AKT activation (Bard-Chapeau et al., 2005).

## Inflammation in Adipose Tissues

In rodents and humans, inflammation in adipose tissues is one mechanism to induce insulin resistance and is mediated by the activation of cellular stress-induced inflammatory signaling pathways. Hyperlipidemia and hyperglycemia caused by excess nutrients, lipolysis, and gluconeogenesis induce mitochondrial dysfunction, ER stress and oxidative stress to stimulate stress responsive signaling molecules such as JNK and IKKβ. In addition to IRS serine-307 phosphorylation, JNK and IKKβ signaling pathways augment inflammatory gene expression in target tissues amplifying systemic inflammation (Samuel and Shulman, 2012). Saturated free fatty acid and gut-derived bacterial lipopolysaccharide (LPS) also bind to Toll-like receptor 4 (TLR4) to activate NF-κB and JNK and mediate inflammation and insulin resistance (Shi et al., 2006; Ghoshal et al., 2009). Furthermore inflammation in adipose tissue is mediated by inflammatory adipokines produced by adipocytes and infiltrated pro-inflammatory immune cells. To summarize the differential adipokine expression and its function in obesity-induced inflammation and insulin resistance, we will focus on the cellular and molecular immune responses in adipose tissues of obese rodents and humans.

Classically in mammals, there are two functional and developmental defined types of adipose tissue, white and brown. Brown adipose tissue is found in newborn humans and hibernating mammals and functionally distinct from white adipose tissue. Brown adipose tissue distributes in cervical-supraclavicular regions in humans and shows polygonal shape with multi-ocular lipid droplets. As the primary function of brown adipose tissue is generating heat, it has a much higher number of mitochondria and capillaries than white adipose tissue (Ravussin and Galgani, 2011). More recently, brown adipose tissue has been identified in humans but there is evidence that this may in fact be a third form of adipose tissue also present in rodent models termed beige or brite adipocytes (Wu et al., 2012a). Similar to brown adipocytes, this recently identified adipocyte subtype is derived from a distinct progenitor (stem) cell population that resides within classical white adipose tissue. In contrast to brown adipocytes, white adipose tissue is well established as an excess energy storage depot as well as an endocrine organ. White adipose tissue is located throughout the body. Subcutaneous and visceral adipose tissues are major adipocyte depots, with additional adipose depots distributed at various organs such as heart, lung, and kidney. Subcutaneous and visceral adipose tissues have differences in gene expression, hypertrophy, and hyperplasia in obesity and differentially contribute to obesity-induced insulin resistance (Hardy et al., 2012). Subcutaneous adipose tissue has high capacity for adipocytes differentiation and cell size expansion to store large amounts of triacylglycerol. This storage capacity serves to reduce visceral adipose tissue mass and lipid deposition in liver and muscle. The inability to convert excess carbohydrate to lipid for storage in subcutaneous adipose tissue (i.e., decreased gene expression such as SREBP-1 and ChREBP) is associated with diabetes in obese humans (Kursawe et al., 2013). In contrast, visceral adipose tissue is positively associated with risk of insulin resistance and shows higher monocytes infiltration and IL-6 production than subcutaneous adipose tissue to induce inflammation in obese subjects (Cancello et al., 2006; Fontana et al., 2007). Ectopic lipid accumulation in liver and muscle is also associated with obesity-induced insulin resistance. High levels of diacylgycerol (DAG) generated by incomplete synthesis to triacylglycerol or breakdown of triacylglycerol to DAG has been proposed to inhibit insulin signaling through protein kinase C activation in muscle (Chin et al., 1994; Griffin et al., 1999; Badin et al., 2013). Similarly, DAG accumulation in the liver is also associated with hepatic insulin resistance (Jornayvaz and Shulman, 2012). In this regard, ATGL deficient mice that have reduced ability to convert triacylglycerol to DAG show enhanced glucose tolerance and insulin sensitivity (Haemmerle et al., 2006). More recently, an alternative model of increased ceramide levels has also been show to associate with insulin resistance (Chavez and Summers, 2012). However, whether DAGs or ceramides mediate a cell autonomous insulin resistance or are part of the complex pathways responsible for obesity-induced inflammation has not been resolved.

**Figure 2:**
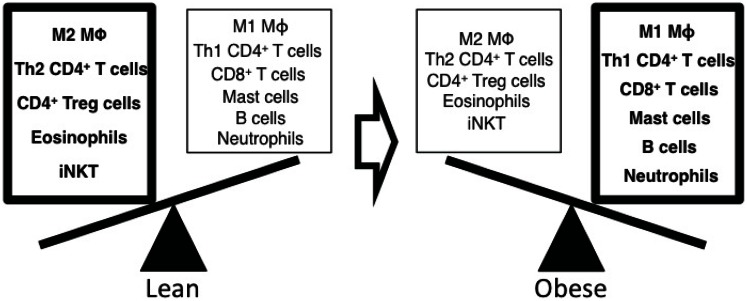
**Altered composition of immune cells with obesity regulates the inflammatory responses in adipose tissue**. Alternatively activated M2 macrophages, Th2 CD4^+^ T cells, regulatory CD4^+^ T cells (T_reg_), eosinophils, and iNKT cells are dominant immune cells in adipose tissue of lean mice. These cells secrete anti-inflammatory cytokines such as IL-4 and IL-10 to suppress inflammation and maintain insulin sensitivity in adipose tissue. In obese mice, the composition of immune cells is dynamically shifted to enhance inflammatory responses in adipose tissue. Classically activated M1 macrophages, Th1 CD4^+^ T cells, effector CD8^+^ T cells, mast cells, B cells, and neutrophils are increased and produce inflammatory mediators such as TNF-α, IFN-γ, autoantibodies, and elastase resulting in insulin resistance.

As eluted to, the inflammatory immune responses in adipose tissues are one of major mechanisms to mediate insulin resistance in rodents and humans, and dynamic changes of immune cell composition in adipose tissues regulate inflammatory responses (Figure 2). White adipose tissue consists of a variety of cell types including adipocytes, macrophages, lymphocytes, fibroblasts, and endothelial cells. Innate immune responses mainly mediated by macrophages generate a key inflammatory process within adipose tissue resulting in insulin resistance. Macrophages differentiate into two functionally distinct populations. Th1 cytokines, IFN-γ, activate nitric-oxide synthase (NOS2) expression in classically activated macrophages (M1), whereas the Th2 cytokines such as IL-4 and IL-13 induce arginase-1 (ARG1) in alternatively activated macrophages (M2) (Mantovani et al., 2004; Lumeng et al., 2007a,b; Mosser and Edwards, 2008; Martinez et al., 2009). F4/80^+^CD206^−^CD11c^+^ inflammatory M1 macrophages are increased in adipose tissue and secrete inflammatory cytokines such as TNF-α, IL-6, and IL-1β. TNF-α levels are increased obese diabetic humans and rodents, and neutralization of TNF-α improves insulin sensitivity in obese rodents (Hotamisligil et al., 1993). TNF-α further enhances the expression of inflammatory cytokines (TNF-α and IL-6) and chemokines (CCL2 and RANTES) in adipocytes. TNF-α also induces serine phosphorylation of IRS1 to modulate the downstream effectors of the insulin receptor resulting in insulin resistance (Hotamisligil et al., 1996). IL-1β is elevated in circulation (Spranger et al., 2003) and in pancreatic islets of obese type 2 diabetic humans and rodents and induces the loss of pancreatic β cell mass resulting in hyperglycemia (Donath et al., 1999; Sauter et al., 2008; Ehses et al., 2009). IL-1β is mainly produced by monocytes and macrophages being synthesized as a IL-1β precursor in the cytosol, and activation-induced NALP3 (cryopyrin) inflammasome activates caspase-1 to mediate active IL-1β secretion (Dinarello, 2009). Thus inflammasome is critical for obesity-induced insulin resistance (Stienstra et al., 2012). In addition, mast cells (Liu et al., 2009), eosinophils (Wu et al., 2011), and dendritic cells (Bertola et al., 2012) are also critically involved in obesity-induced inflammation and insulin resistance through the production of pro- and anti-inflammatory cytokines in adipose tissues.

Neutrophils are the first immune cells to respond to inflammation and involved in the trafficking of other immune cells into inflammatory sites. Neutrophils quickly infiltrate into adipose tissue and produce neutrophil elastase, which accelerates inflammatory responses. Thus deletion of neutrophil elastase shows less inflammation and improved insulin sensitivity in obese rodents with reduced neutrophils and macrophages in adipose tissues (Talukdar et al., 2012). Recently the role of innate invariant natural killer T (iNKT) cells in obesity-induced insulin resistance has been shown. As iNKT cells rapidly response to its ligands, iNKT cells generate Th1 and Th2 cytokines including IFN-γ and IL-4 to quickly regulate immune responses (Bendelac et al., 2007). iNKT cells also produce IL-17 after TGF-β and IL-1β stimulation resulting in neutrophilic airway inflammation (Monteiro et al., 2013). iNKT cells are highly enriched in adipose tissue of lean rodents and humans. However iNKT cells are decreased in adipose tissues of obese rodents and humans, and the number of iNKT cells is recovered after weight loss. iNKT cell deficient mice show that iNKT cells protect inflammation and insulin resistance in both lean and obese rodents and humans as adipose tissue-derived iNKT cells produce anti-inflammatory cytokines (Lynch et al., 2012; Schipper et al., 2012). However, the role of iNKT cells in HFD-induced inflammation and insulin resistance is still controversial as several previous reports show that iNKT cells are not necessary to suppress the HFD-induced inflammation and insulin resistance (Mantell et al., 2011; Wu et al., 2012b).

Adaptive immune responses have been also shown to be a critical factor for HFD-induced inflammation and insulin resistance in humans and rodents. CD4^+^ T cells in adipose tissues of obese rodents and humans mediate HFD-induced insulin resistance. IFN-γ producing Th1 CD4^+^ T cells are increased in adipose tissues of obese mice overwhelming the anti-inflammatory Th2 CD4^+^ T cells and Foxp3^+^ regulatory CD4^+^ T cells. Interestingly, adoptive transfer of CD4^+^ cells especially Th2 CD4^+^ T cells, which produce IL-4 and IL-13, rescues HFD-induced obesity and insulin resistance in *Rag1* deficient mice suggesting that Th2 cytokines such as IL-4 and IL-13 suppress HFD-induced inflammation to improve insulin sensitivity (Winer et al., 2009a). Foxp3^+^CD4^+^ regulatory T cells (T_reg_), anti-inflammatory IL-10 producer, are unique cell population that suppresses inflammation, and T_reg_ cells are decreased in HFD-induced and genetically modified obese mice resulting in insulin resistance (Feuerer et al., 2009). Cytotoxic CD8^+^ T cells are also significantly increased in adipose tissues of obese mice, and depletion of CD8^+^ T cells reverses inflammation and insulin resistance suggesting that obesity-induced infiltration of CD8^+^ T cells deteriorate systemic insulin sensitivity (Nishimura et al., 2009). Although inflammatory Th17 CD4^+^ T cells mediate diverse autoimmune diseases, and HFD predisposes autoimmune diseases such as trinitrobenzene sulfonic acid (TNBS) colitis and experimental autoimmune encephalomyelitis (EAE) (Winer et al., 2009b), Th17 CD4^+^ T cells are not involved in the inflammation of obese mice (Winer et al., 2009a). B cells are also critical for the development of inflammation in adipose tissues. B cells are accumulated in adipose tissues of obese rodents and induce inflammation and insulin resistance along with macrophages and T cells. B cells produce IgG2c autoantibodies to induce systemic inflammation, and B cell deficient mice and depletion of B cells using anti-CD20 antibody administration suppress systemic inflammation and enhance insulin sensitivity (Winer et al., 2011).

## Adipokines

It is now well established that white adipose tissue functions as an active endocrine organ to modulate physiological metabolic processes. As adipose tissue contains various cell types such as adipocytes, immune cells, endothelial cells, and fibroblasts, it produces and releases diverse secretory proteins called adipokines into the systemic circulation. Visceral and subcutaneous adipose tissues produce unique profiles of adipokines to mediate inflammation and insulin resistance in obese rodents and humans. Two decades ago, adipsin (complement factor C) (Cook et al., 1987), TNF-α, and leptin were identified as bona fide adipokines, and those studies facilitate finding other secreted adipocyte factors. Although adipokines have multiple metabolic functions, we will mainly discuss the inflammatory functions of adipokines that play important roles in mediating obesity-induced insulin resistance. In this regard, adipokines are classified as pro- and anti-inflammatory adipokines according to their effects on inflammatory responses in adipose tissues. Most adipokines show pro-inflammatory activity with the noted exceptions of adiponectin, secreted frizzled-related protein 5 (SFRP5), visceral adipose tissue-derived serine protease inhibitor (Vaspin), and omentin-1. The pro-inflammatory adipokines are increased whereas the anti-inflammatory adipokines are decreased in obese rodents and humans that are associated with insulin resistance.

## Pro-Inflammatory Adipokines

### Leptin

Obese mutation *ob*, an autosomal recessive mutation, increases food intake and obesity resulting in T2D. Para-biosis experiments show that *Lep^*ob*^/Lep^*ob*^* mice have defects in circulating factor(s), which regulate food intake. Leptin expressed from obese gene is identified by positional cloning and has been shown to regulate feeding behavior through the hypothalamic regulation in central nervous system (Zhang et al., 1994). Thus leptin deficient *Lep^*ob*^/Lep^*ob*^* and leptin receptor deficient *Lepr^*db*^/Lepr^*db*^* mice display marked hyperphagia, obesity, and insulin resistance. Importantly, exogenous administration of leptin to *Lep^*ob*^/Lep^*ob*^* mice reduces obesity and restores insulin sensitivity. However, leptin levels in circulation are increased in obese rodents and humans suggesting that obese subjects display leptin resistance (Friedman and Halaas, 1998). Leptin resistance has been reported as mediated by impaired leptin transport in blood brain barrier, hyperleptinemia-induced SOCS3 (Kievit et al., 2006), defective autophagy (Quan et al., 2012), and ER stress (Ozcan et al., 2009). Obesity-induced chronic inflammation also induces leptin resistance through the activation of TLR4, JNK, and IKKβ (Zhang et al., 2008; Kleinridders et al., 2009).

The structure of leptin is similar to pro-inflammatory helical cytokines including IL-2, IL-6, and granulocyte-colony stimulating factor (G-CSF), and leptin indeed induces inflammatory responses through the long isoform of the leptin receptor b (LepR_b_) and its proximal Janus kinase 2 (JAK2) and signal transducer and activator of transcription 3 (STAT3) signaling pathway. Leptin activates monocytes and macrophages to produce pro-inflammatory IL-6, TNF-α, and IL-12 (Gainsford et al., 1996) and stimulates the production of CCL2 and vascular endothelial growth factor in human hepatic stellate cells (Aleffi et al., 2005). Other inflammatory signals such as TNF-α and LPS stimulate the expression of leptin and leptin receptor (Grunfeld et al., 1996; Gan et al., 2012). Leptin also enhances the production of pro-inflammatory Th1 cytokines whereas suppresses the production of anti-inflammatory Th2 cytokines such as IL-4 in CD4^+^ T cells (Lord et al., 1998). Thus *Lep^*ob*^/Lep^*ob*^* and *Lepr^*db*^/Lepr^*db*^* mice are resistant to Con-A induced hepatitis and EAE as *Lep^*ob*^/Lep^*ob*^* and *Lepr^*db*^/Lepr^*db*^* mice are skewed to an anti-inflammatory Th2 immune response due to the lack of leptin signaling (Faggioni et al., 2000; Matarese et al., 2001). Furthermore, leptin induces collagen-induced arthritis through the differentiation of Th17 CD4^+^ T cells to enhance joint inflammation (Deng et al., 2012).

### Interleukin-6

The role of IL-6 in obesity and insulin resistance is controversial. IL-6 is highly expressed in adipose tissue and positively correlated with obesity in humans. Peripheral administration of IL-6 interrupts insulin signaling due to enhance expression of SOCS3 in hepatocytes suggesting that obesity-induced IL-6 expression mediates insulin resistance (Senn et al., 2003). In contrast, IL-6 deficient mice show mature-onset obesity and hepatic inflammation, and IL-6 administration reverses insulin resistance (Wallenius et al., 2002; Matthews et al., 2010). As central administration of IL-6 enhances energy expenditure and decreases obesity, IL-6 can also influence obesity and insulin sensitivity through a central nervous system mechanism. Thus the role IL-6 in obesity and insulin resistance likely depends upon the specific sites of expression that is integrated with other adipokine/cytokine factors in a systems integrated manner.

### Tumor necrosis factor

TNF-α was originally identified as an endotoxin-induced serum factor that mediates tumor necrosis and cancer cachexia. TNF-α is mainly expressed in monocytes and macrophages as a 26 kDa transmembrane protein and then is converted to active trimer by TNF-α converting enzyme. TNF-α is a typical pro-inflammatory cytokine that is increased in obese humans and rodents suggesting that TNF-α contributes to insulin resistance. TNF-α treatment in cell lines and rodents induces insulin resistance, and neutralization of TNF-α in obese *fa*/*fa* rats enhances insulin sensitivity (Hotamisligil et al., 1993). Accordingly TNF-α or its receptors deficient mice show improved insulin sensitivity in white adipose tissues and skeletal muscles of HFD fed and *Lep^*ob*^/Lep^*ob*^* mice (Uysal et al., 1997). TNF-α stimulates the phosphorylation of IRS on Ser-307 residues that suppresses insulin-induced IRS1 tyrosine phosphorylation and activation of down stream targets (Hotamisligil et al., 1996). Although TNF-α levels in the circulation is positively correlated with insulin resistance, and neutralization of TNF-α improved the insulin sensitivity in rodents, clinical effects of TNF-α neutralization in humans are still controversial. Short-term administration of TNF-α blocking reagents to obese T2D patients suppresses inflammation but dose not show improved insulin sensitivity (Ofei et al., 1996). In contrast, long-term treatment of TNF-α blocking reagents in obese patients with severe inflammatory diseases such as rheumatoid arthritis improves insulin sensitivity (Gonzalez-Gay et al., 2006; Stanley et al., 2011). TNF-α also suppresses the expression of phosphodiesterase 3B (PDE3B) and perilipin. As PDE3B reduces cAMP after insulin stimulation, and perilipin regulates the access of hormone-sensitive lipase in adipocytes, TNF-α induces lipolysis in adipocytes to release free fatty acid (Souza et al., 1998; Zhang et al., 2002). Free fatty acid in turn binds to TLR4, and pro-inflammatory factors are expressed through NF-κB activation (Lee et al., 2001). Consistent with this model, TLR4 deficient mice show improved HFD-induced insulin resistance (Kim et al., 2007a).

### Retinol binding protein 4

Retinol binding protein 4 expressed in liver, adipocytes, and macrophages is significantly increased in obese diabetic rodents and humans. The expression of RBP4 is inversely correlated with that of GLUT4 in adipocytes, and administration of recombinant RBP4 to normal mice induces insulin resistance (Yang et al., 2005). RBP4 inhibits insulin-induced phosphorylation of IRS1 suggesting that adipocyte secreting RBP4 induces insulin resistance. Clinical studies show that increased RBP4 levels are closely associated with high blood pressure, high levels of triacylglycerol, high body mass index (BMI) (Graham et al., 2006), subclinical inflammation, and nephropathy (Akbay et al., 2010). In fact, RBP4 stimulates human primary endothelial cells to produce pro-inflammatory molecules such as vascular cell adhesion molecule 1 (VCAM1), CCL2, and IL-6 resulting in the progression of endothelial inflammation in cardiovascular disease and microvascular complication in diabetes (Farjo et al., 2012).

### Resistin

Resistin (ADSF/FIZZ3/XCP1), 10 kDa polypeptide with 114 amino acids in rodents, is identified as an inducer of pulmonary inflammation (Holcomb et al., 2000) and insulin resistance (Steppan et al., 2001). Resistin belongs to the cysteine-rich family and circulates as a hexamer and trimer. High molecular weight hexamer is more abundant but less active than trimer that strongly induces insulin resistance. Resistin is involved in the activation of SOCS3 resulting in the suppression of insulin-mediated signaling in adipocytes (Steppan et al., 2005). Thus resistin deficient *Lep^*ob*^/Lep^*ob*^* mice show improved glucose tolerance and insulin sensitivity (Qi et al., 2006). In contrast the function of resistin in humans is not clear, as resistin levels in blood circulation are not correlated with obesity and insulin resistance. Monocytes and macrophages are major sources of resistin in humans although the expression of resistin is restricted in adipocytes in rodents. Inflammatory cytokines such as IL-1β, IL-6, TNF-α, and LPS induce the resistin expression in human macrophages. Resistin stimulates human peripheral mononuclear cells to produce IL-6 and TNF-α through the NF-κB signaling pathway, and rosiglitazone a PPARγ agonist suppresses the resistin expression in adipose tissues resulting in the attenuation of inflammatory responses (Bokarewa et al., 2005). Resistin also activates JNK and p38 MAPK to induce insulin resistance through TLR4 binding in the hypothalamus (Benomar et al., 2013).

### CC-chemokine ligand 2 and CC-chemokine receptor type 5

Chemokines and their receptors play essential roles in mediating infiltration of immune cells into adipose tissue. CCL2 (MCP1) and CCR5 are typical chemokine and chemokine receptor, respectively that mediate inflammatory responses and are significantly enhanced in obese rodents and humans. Accordingly CCR2, the receptor of CCL2, deficient mice show attenuated macrophage infiltration, inflammation, and insulin resistance (Weisberg et al., 2006). In addition, genetic deletion of a related receptor CCR5 has recently been shown to improve inflammation, insulin sensitivity, and hepatic steatosis with reduced macrophage infiltration and preferred anti-inflammatory M2 macrophage differentiation in obese mice (Kitade et al., 2012). However the role CCL2 in inflammation and insulin resistance is not clear. In one study CCL2 deficient mice show decreased macrophage infiltration and inflammation in adipose tissues (Kanda et al., 2006) whereas in another study CCL2 deficient mice show no differences in macrophage accumulation and inflammation in adipose tissue of obese mice (Kirk et al., 2008). Although the basis for this difference is not known, it is possible that CCL2 deficiency might be compensated by other related chemokines in certain genetic background.

### Angiopoietin-like protein 2

Adipose tissue is the primary source of angiopoietin-like protein 2 (ANGPTL2), and ANGPTL2 expression is enhanced in obese humans and rodents (Tabata et al., 2009). ANGPTL2 has the N-terminal coiled coil domain for oligomerization and the C-terminal fibrinogen-like domain. ANGPTL2 activates endothelial cells and macrophages to increase inflammatory responses through integrin mediated signaling. Thus ANGPTL2 deficiency ameliorates inflammation and insulin resistance in HFD fed mice. In addition, inflammatory cytokines such as TNF-α induce the expression of ANGPTL2 in 3T3-L1 adipocytes through PI3K-FOXO1 activation (Zheng et al., 2011).

### Chemerin

Chemerin is a ligand of the G protein-coupled receptor ChemR23 (Wittamer et al., 2003) and expressed in most tissues except leukocytes. Chemerin mediates inflammatory responses, as it is a chemoattractant to induce the infiltration of macrophages, immature dendritic cells, and NK cells in inflammatory disease such as ulcerative colitis and skin lupus (Albanesi et al., 2009). In addition chemerin has been shown as an adipokine to regulate adipogenesis and adipocytes metabolism (Goralski et al., 2007) although molecular mechanisms are still controversial (Bondue et al., 2011). Chemerin level is positively correlated with BMI, fasting glucose, triacylglycerols, and inflammatory cytokines in obese subjects, and administration of chemerin exacerbates glucose intolerance in obese mice (Ernst et al., 2010). However, chemerin suppresses the zymosan-induced peritonitis suggesting that chemerin also has anti-inflammatory activity (Cash et al., 2008).

## Anti-Inflammatory Adipokines

### Adiponectin

Adiponectin is highly expressed by adipocytes with potent anti-inflammatory properties. Adiponectin has an N-terminal collagen-like domain and a C-terminal complement factor C1q-like globular domain and circulates as trimers, hexamers, and a high molecular weight form. As pro-inflammatory factors such as TNF-α, IL-6, ROS, and hypoxia suppress the expression of adiponectin in adipocytes, adiponectin levels are decreased in obese rodents and humans (Li et al., 2009). Recently it has been shown that not only inflammatory signals but iron overload in adipocytes suppresses adiponectin expression in obese humans through FOXO1 (Gabrielsen et al., 2012). In contrast PPARγ antagonists stimulate the expression of adiponectin in adipocytes (Maeda et al., 2001). Adiponectin activates AMP-dependent protein kinase (AMPK) through its receptors, ADIPOR1/2, to enhance fatty acid oxidation and glucose uptake in muscle and to suppress gluconeogenesis in liver (Yamauchi et al., 2002). Exogenous administration of adiponectin or overexpression in transgenic mice results in improved insulin sensitivity whereas adiponectin deficient mice develop HFD-induced inflammation and insulin resistance (Maeda et al., 2002; Kim et al., 2007b). Adiponectin inhibits LPS-induced TNF-α production in macrophages through inhibition of NF-κB activation and stimulate the production of anti-inflammatory IL-10 (Yokota et al., 2000; Kumada et al., 2004). Adiponectin also promotes the differentiation of anti-inflammatory M2 macrophages and phagocytosis to remove apoptotic cells (Takemura et al., 2007). Adiponectin modulates T cells activation and inflammatory function of NK cells. Adiponectin receptors are upregulated on the surface of human T cells after antigen stimulation and mediate apoptosis of antigen specific T cells resulting in the suppression of antigen specific T cells expansion (Wilk et al., 2011). Furthermore adiponectin suppresses TLR-mediated IFN-γ production in NK cells without affecting in cytotoxicity of NK cells (Wilk et al., 2013). Thus adiponectin can also suppresses the development of atherosclerosis, fatty liver diseases, and liver fibrosis (Okamoto et al., 2002; Xu et al., 2003).

### Secreted frizzled-related protein 5

Secreted frizzled-related protein has an N-terminal cysteine-rich domain that is homologous to frizzled proteins, the cell surface receptors for wingless-type MMTV integration site family (WNT). Thus SFRP5 that is highly expressed in adipocytes of mouse white adipose tissues prevents the binding of WNT proteins to its receptors. WNT proteins especially WNT5a is closely linked inflammatory responses. The expression of SFRP5 is decreased, but the expression of WNT5a, an antagonizing target of SFRP5, is increased in white adipose tissues of obese rodents and humans suggesting that SFRP5 might have potential to attenuate inflammatory effect of WNT5a in adipose tissues. Accordingly, HFD fed SFRP5 deficient mice *Sfrp*^−*/*−^ have insulin resistance and fatty liver along with enhanced inflammatory macrophage accumulation to produce IL-6, TNF-α, and CCL2 suggesting that SFRP5 is an anti-inflammatory adipokine (Ouchi et al., 2010). WNT5a induces the non-canonical activation of JNK1, and SFRP5 deficient mice show highly activated JNK1 in HFD indicating that SFRP5 inhibits WNT5a mediated non-canonical JNK1 activation in adipose tissues to suppress obesity-induced inflammation and insulin resistance. In contrast, another SFRP5 deficient mice *Sfrp5*^*Q*27s*top*^ recently show that SFRP5 expression is increased in obese mice, and SFRP5 enhances adipogenesis as SFRP5 suppresses WNT signaling (Mori et al., 2012).

### Visceral adipose tissue-derived serine protease inhibitor

Visceral adipose tissue-derived serine protease inhibitor is identified from visceral white adipose tissues of Otsuka Ling-Evans Tokushima fatty (OLETF) rat as an insulin sensitizing adipokine because vaspin suppresses the expression of pro-inflammatory adipokines such as resistin, leptin, and TNF-α (Hida et al., 2005). Vaspin is highly expressed by rat adipocytes and improves insulin sensitivity whereas the effect of vaspin in humans is still unclear. Inflammatory stimulators including TNF-α play an important role in the development of atherosclerosis. Vaspin also suppresses TNF-α-induced ROS production and monocytes adhesion to smooth muscle cells by inhibiting the activation of NF-κB and PKCθ (Phalitakul et al., 2011).

### Omentin-1

Human omental adipose tissues secrete omentin-1 that is preferentially expressed by omental stromal vascular fraction cells, but not by adipocytes (Schaffler et al., 2005). Omentin-1 levels in blood circulation are inversely related with obesity and suppressed by glucose and insulin (de Souza Batista et al., 2007). Omentin-1 enhances the insulin-induced glucose uptake in human visceral and subcutaneous adipocytes through increased phosphorylation of AKT/PKB (Yang et al., 2006). Interestingly, omentin-1 attenuates C-reactive protein (CRP) and TNF-α-induced NF-κB activation in human endothelial cells suggesting that omentin-1 might be an anti-inflammatory adipokine in humans (Tan et al., 2010).

### Apelin

Apelin expressed in many tissues such as lung, mammary gland, and testis is identified as the endogenous ligand of orphan G protein-coupled receptor termed APJ (Tatemoto et al., 1998). Apelin has diverse physiological functions to regulate fluid homeostasis, heart rate, and metabolic functions (Carpene et al., 2007). Adipocytes produce apelin, and its plasma level is increased in obese humans and rodents. As apelin enhances glucose uptake through AMPK-dependent manner and suppresses lipolysis, apelin deficient mice show insulin resistance following HFD feeding (Yue et al., 2010, 2011) suggesting that apelin improves glucose homeostasis and insulin sensitivity. Apelin is also involved in inflammatory responses in obese subjects. Apelin expression is positively associated with TNF-α, and TNF-α treatment induces the apelin expression in adipose tissue. In addition, apelin activates JNK and NF-κB to induce inflammatory adhesion molecules such as ICAM in human umbilical vein endothelial cells (Lu et al., 2012). However, apelin administration reduces inflammation in kidney to ameliorate diabetic nephropathy through the suppression of CCL2 expression, monocytes infiltration, and NF-κB activation (Day et al., 2013). Thus the precise role for apelin in regulating inflammatory responses remains undefined.

## Inflammation and Insulin Resistance

As described above, in both rodents and humans obesity-related insulin resistance is strongly associated with a relative increase in inflammation in adipose tissue. Numerous genetic mouse models have clearly demonstrated that prevention against this pro-inflammatory response protects against diet-induced insulin resistance but not against obesity (Kim et al., 2008). Moreover, in humans approximately 20% of the obese population remains fully insulin sensitive and metabolically normal, termed metabolically benign obesity (Ferrannini et al., 1997). Although the insulin resistant obese population displays adipose tissue inflammation, the metabolically benign obese population has a similar adipose tissue inflammatory cytokine, adipokine, and immune cell distribution as normal insulin sensitive non-obese individuals (Karelis et al., 2005). Recent clinical studies have also shown that treatment with salsalate a non-steroidal anti-inflammatory drug (NSAID) derived from salicylate improves insulin sensitivity in obese insulin resistant patients (Goldfine et al., 2010, 2013).

Despite these accumulating data supporting adipose tissue inflammation as a causative factor in diet-induced insulin resistance, it remains unclear what is the initiation factor(s) that is responsible for generating the adipose tissue inflammatory cascade. It has been suggested that adipocyte released chemokines such as CCL2 is responsible for the initiation of pro-inflammatory macrophage infiltration (Weisberg et al., 2006). However, we now know that infiltration of macrophages is a late step in the adipose tissue inflammatory process and that one of the earliest events is the infiltration of neutrophils (Elgazar-Carmon et al., 2008; Talukdar et al., 2012). Whether or not neutrophil recruitment/activation in adipose tissue is the initiator of the inflammatory cascade and/or the signals responsible for neutrophil recruitment remain undetermined.

In any case, another unresolved issue is the mechanisms by which adipose tissue inflammation results in liver and skeletal muscle insulin resistance. Several studies have also observed the local liver and skeletal muscle expression of pro-inflammatory cytokines and activation of inflammatory cells (Odegaard and Chawla, 2013). Whether this results from systemic inflammation emanating from adipose tissue or is due to a local release of pro-inflammatory chemokines/cytokines has yet to be established. Moreover, whether this accounts for the hepatic and skeletal muscle insulin resistance or results from alterations in central signaling and/or systemic factors is area that needs further study.

It is becoming clear that targeting the pro-inflammatory pathway may provide a novel therapeutic approach to prevent insulin resistance, particularly in obesity-induced insulin resistance. For example, although early efforts to block TNF-α failed to show efficacy, more recently the use of salicylate to reduce inflammation by inhibiting IKKβ signaling was found to improve insulin sensitivity in animal models and humans (Goldfine et al., 2010, 2013). More recently, amlexanox, an inhibitor of the non-canonical IκB kinases IKK-ε and TANK-binding kinase 1 (TBK1) was shown to not only improve sensitivity but to also increase energy expenditure and weight loss in obese mice (Reilly et al., 2013). These data provide the proof of principal that targeting the inflammatory signaling pathway can be an effective approach in the treatment of insulin resistance.

## Perspectives

In summary, adipose tissue has multiple integrative functions serving as energy storage organ that can provide fuel for energy production in times of external nutrient shortage. However, over the past two decades adipocytes have become established as bona fide professional endocrine cells that integrates whole body energy status with eating behavior, energy expenditure, and insulin sensitivity. Moreover, adipose tissue has become a central node for driving local and systemic sterile inflammation that is a key element in obesity-induced insulin resistance. Although many adipokines have been identified and well studied, the identification and functional studies of new adipokines and their control of integrative physiologic responses are essential to understand pathophysiological mechanisms of obesity-induced metabolic diseases.

## Conflict of Interest Statement

The authors declare that the research was conducted in the absence of any commercial or financial relationships that could be construed as a potential conflict of interest.
